# Supplementary material for: Pterodon emarginatus Seed Preparations: Antiradical Activity, Chemical Characterization, and In Silico ADMET Parameters of β-caryophyllene and Farnesol
Source: Molecules. 2023 Nov 9;28(22):7494. doi: 10.3390/molecules28227494 (PMC10673522; doi:10.3390/molecules28227494)
Supplement: Supplementary file 1 [file molecules-28-07494-s001.zip › molecules-2694899-supplementary.pdf]

molecules

Supplementary material

Pterodon emarginatus Seed Preparations: Antiradical Activity, Chemical Characterization, and In Silico ADMET Parameters of β-caryophyllene and Farnesol

Guglielmina Froldi [1,*], Francesco Benetti [1], Andrea Mondin [2], Marco Roverso [2], Elisa Pangrazzi [1], Francine Medjiofack Djeujo [1] and Paolo Pastore [2]

[1] Department of Pharmaceutical and Pharmacological Sciences, University of Padova, 35131 Padova, Italy; francesco.benetti90@gmail.com (F.B.); elisapangrazzi99@gmail.com (E.P.); francine.medjiofackdjeujo@phd.unipd.it (F.M.D.)
[2] Department of Chemical Sciences, University of Padova, 35131 Padova, Italy; mondin.andrea@gmail.com (A.M.); marco.roverso@unipd.it (M.R.); paolo.pastore@unipd.it (P.P.)
* Correspondence: g.froldi@unipd.it; Tel.: +39-049-827-5092; Fax: +39-049-827-5093

Abstract: The study of medicinal plants and their active compounds is relevant to maintaining knowledge of traditional medicine and to the development of new drugs of natural origin with lower environmental impact. From the seeds of the Brazilian plant *Pterodon emarginatus,* six different preparations were obtained: essential oil (EO), ethanol extract (EthE) prepared using the traditional method, and four extracts using solvents at different polarities, such as n-hexane, chloroform, ethyl acetate, and methanol (HexE, ChlE, EtAE, and MetE). Chemical characterization was carried out with gas chromatography, allowing the identification of several terpenoids as characteristic components. The two sesquiterpenes β-caryophyllene and farnesol were identified in all preparations of *Pterodon emarginatus,* and their amounts were also evaluated. Furthermore, the total flavonoid and phenolic contents of the extracts were assessed. Successively, the antiradical activity with DPPH and ORAC assays and the influence on cell proliferation by the MTT test on the human colorectal adenocarcinoma (HT-29) cell line of the preparations and the two compounds were evaluated. Lastly, an in silico study of adsorption, distribution, metabolism, excretion, and toxicity (ADMET) showed that β-caryophyllene and farnesol could be suitable candidates for development as drugs. The set of data obtained highlights the potential medicinal use of *Pterodon emarginatus* seeds and supports further studies of both plant preparations and isolated compounds, β-caryophyllene and farnesol, for their potential use in disease with free radical involvement as age-related chronic disorders.

Keywords: phenols; terpenes; medicinal plants; ADMETlab web tool; essential oil; traditional medicine; gas chromatography; antioxidants; DPPH assay; ORAC assay; ADMET

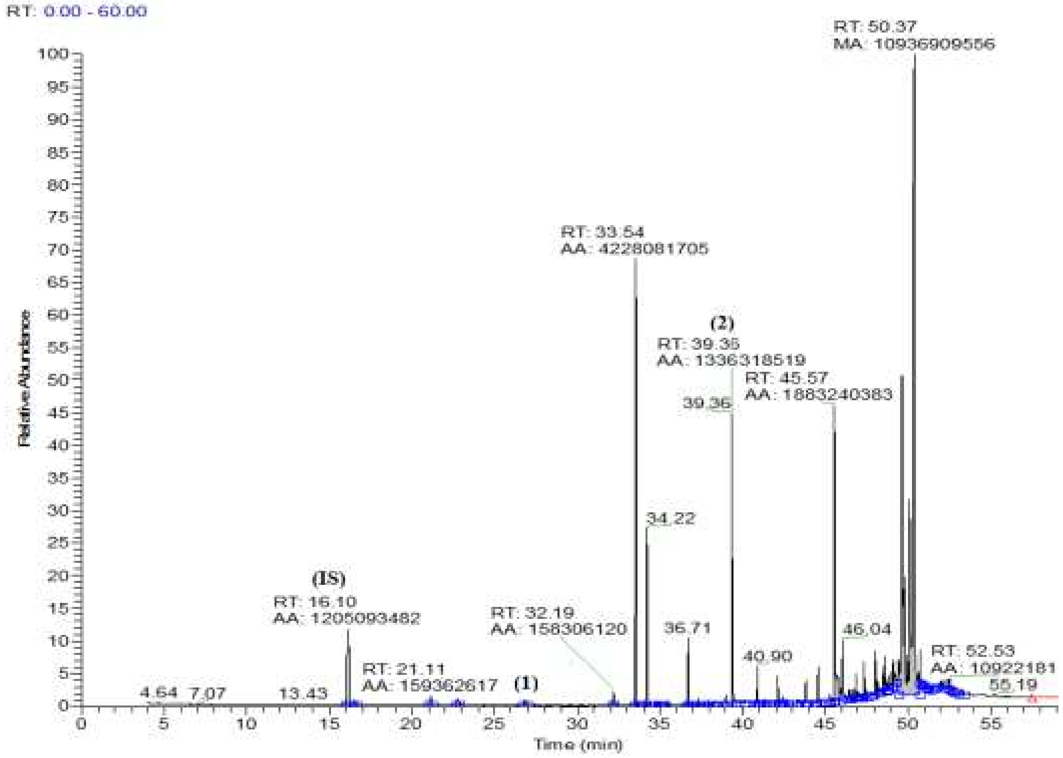

Figure S1 An example of a gas chromatogram of ethanol extract (EthE) obtained from the seeds of *Pterodon emarginatus*. IS: 1,3,5-triisopropylbenzene. (1): β-caryophyllene; (2): farnesol.

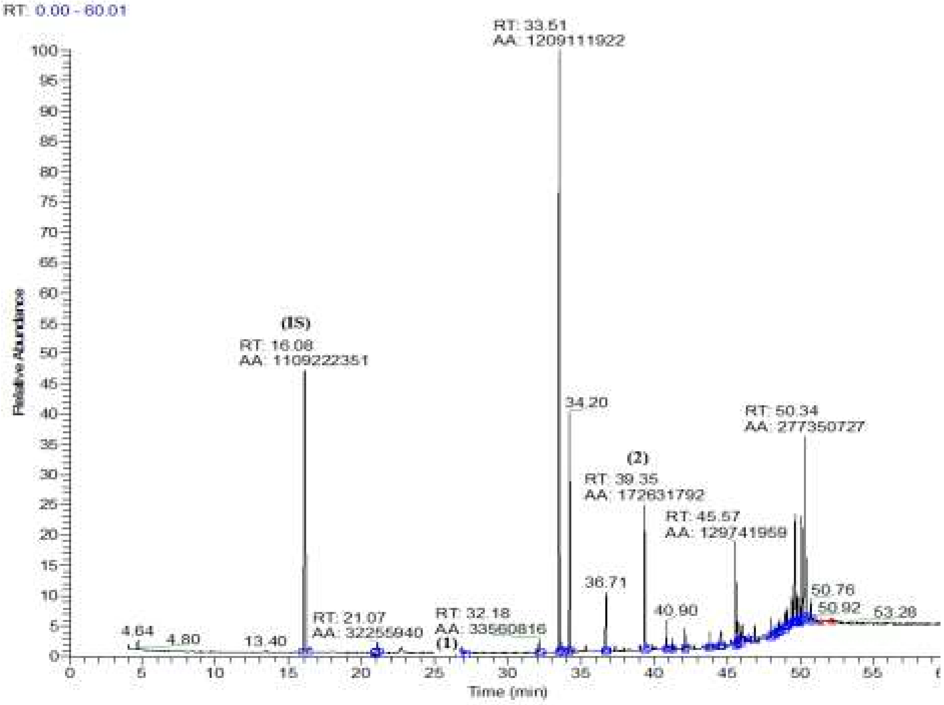

Figura S2 An example of a gas chromatogram of hexane extract (HexE) obtained from the seeds of *Pterodon emarginatus*. IS: 1,3,5-triisopropylbenzene. (1): β-caryophyllene; (2): farnesol.

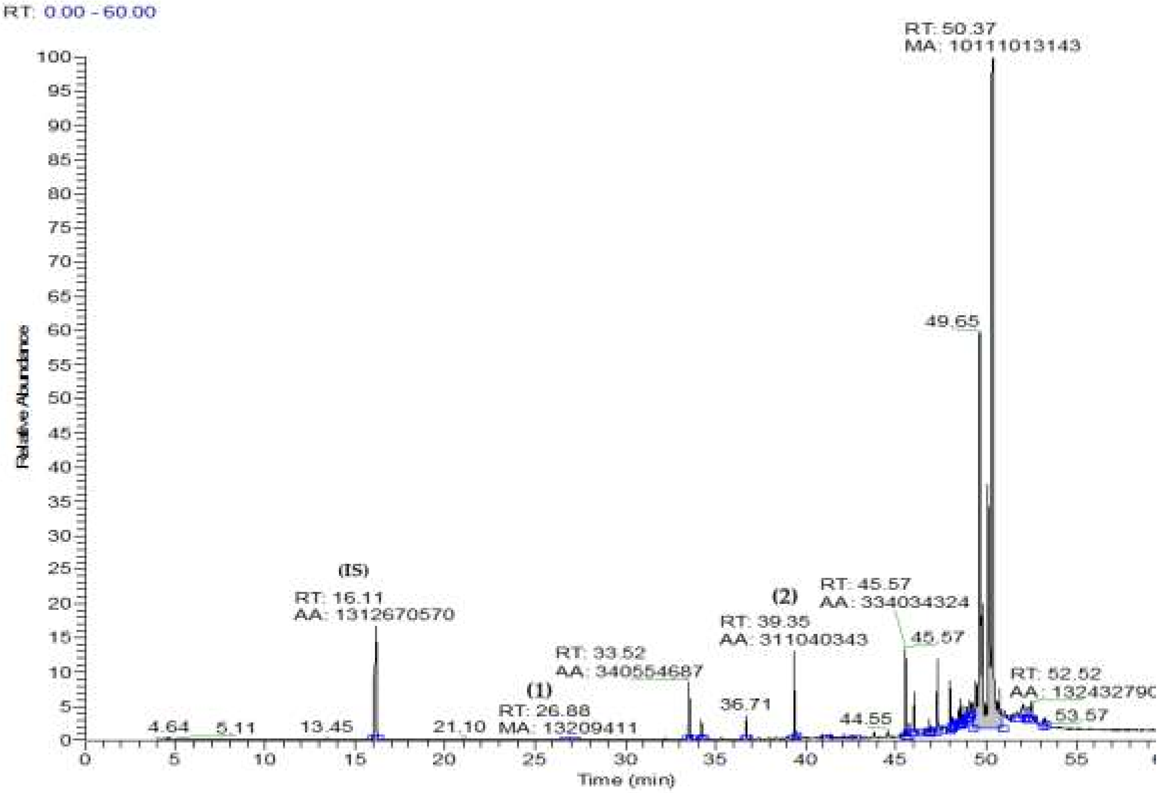

Figure S3 An example of a gas chromatogram of chloroform extract (ChlE) obtained from the seeds of *Pterodon emarginatus*. IS: 1,3,5-triisopropylbenzene. (1): β-caryophyllene; (2): farnesol.

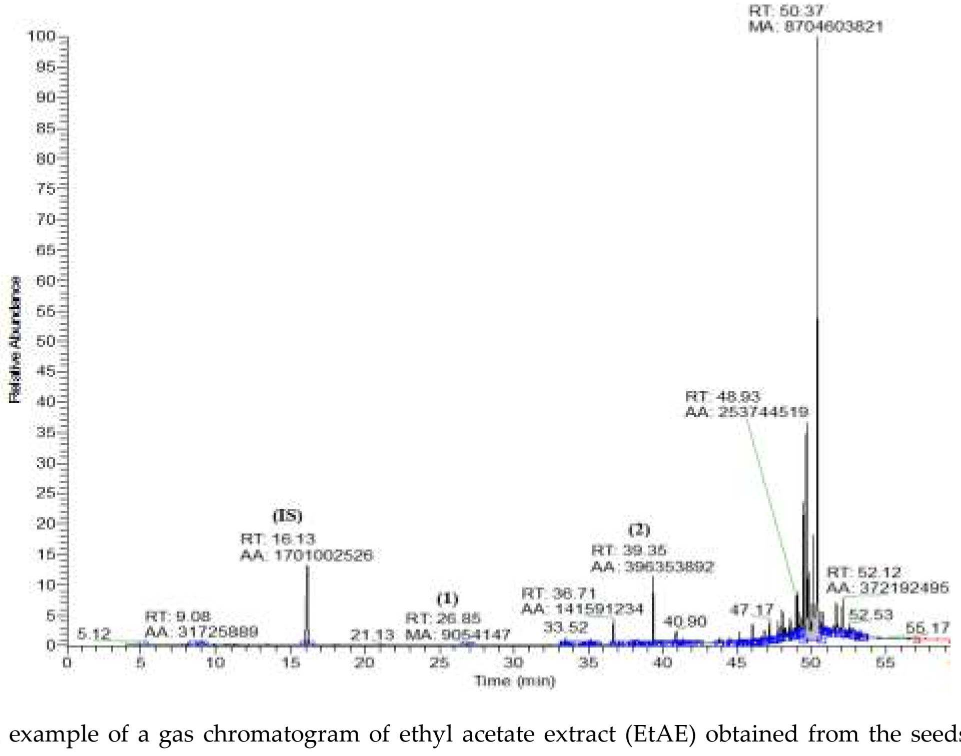

Figure S4 An example of a gas chromatogram of ethyl acetate extract (EtAE) obtained from the seeds of *Pterodon emarginatus*. IS: 1,3,5-triisopropylbenzene. (1): β-caryophyllene; (2): farnesol.

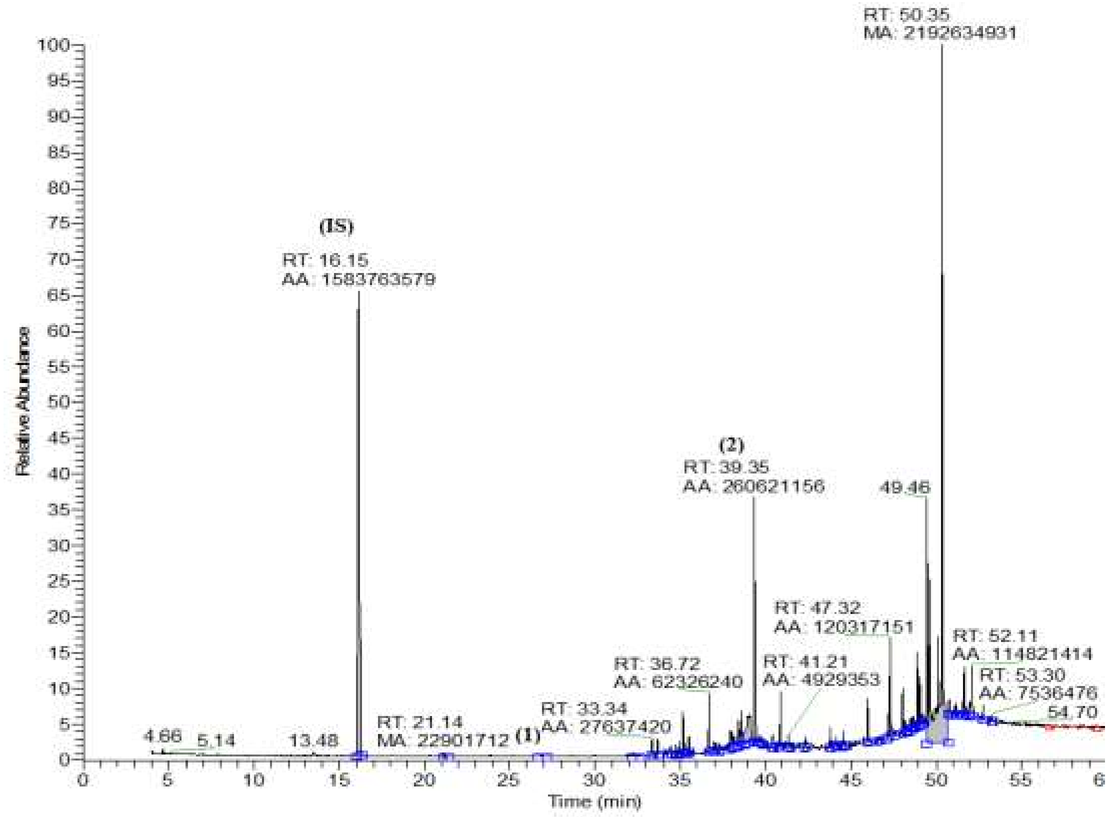

Figure S5 An example of a gas chromatogram of methanol extract (MetE)) obtained from the seeds of *Pterodon emarginatus*. IS: 1,3,5-triisopropylbenzene. (1): β-caryophyllene; (2): farnesol.

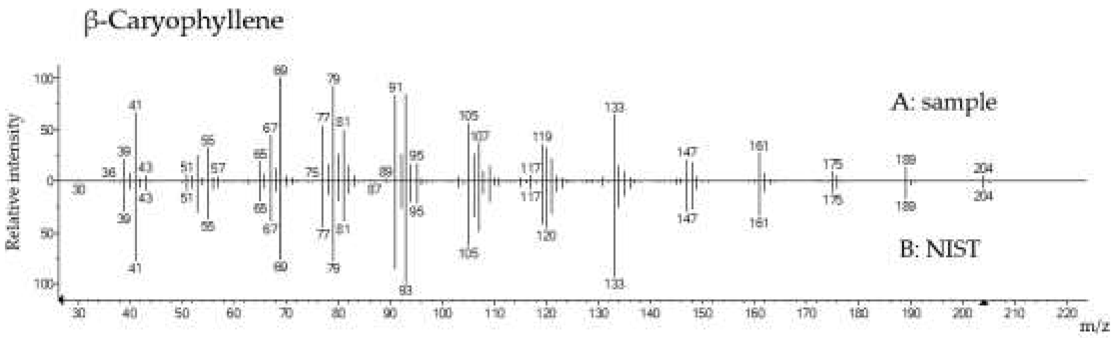

Figure S6 An example of comparing the mass spectrum of β-caryophyllene (Rt = 26.88 min EO) in *Pterodon emarginatus* samples (panel A) and the NIST database (panel B). Match and Reverse Match exceeded 890 in all samples.

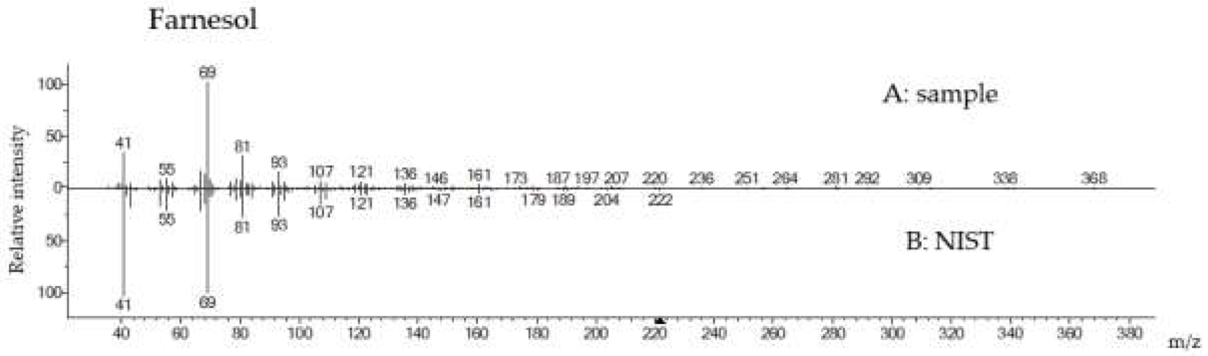

Figure S7 An example of comparing the mass spectrum of farnesol (Rt = 39.35 min EO) in *Pterodon emarginatus* samples (panel A) and the NIST database (panel B). Match and Reverse Match exceeded 890 in all samples.

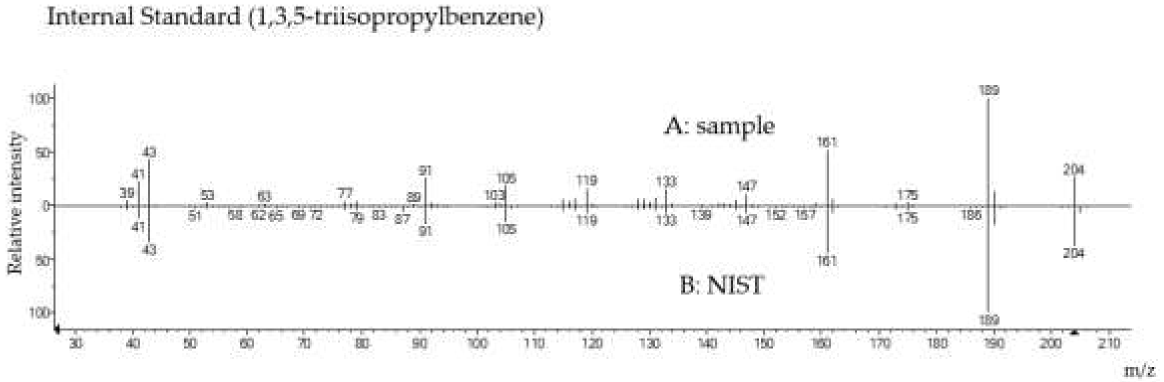

Figure S8 An example of comparing the mass spectrum of Internal Standard (1,3,5-triisopropylbenzene, Rt = 16.11 min EO) in *Pterodon emarginatus* samples (panel A) and the NIST database (panel B). Match and Reverse Match exceeded 890 in all samples.

α-Copaene

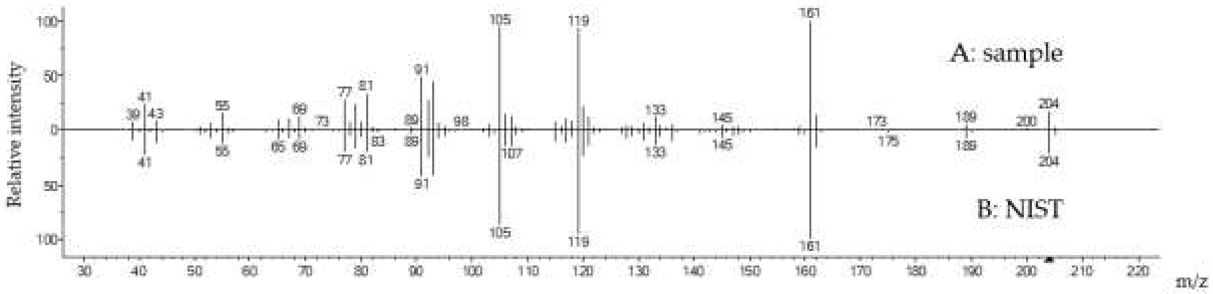

Figure S9 An example of comparing the mass spectrum of α-copaene (Rt = 21.12 min EO) in *Pterodon emarginatus* samples (panel A) and the NIST database (panel B). Match and Reverse Match exceeded 890 in all samples.

Allo-aromadendrene

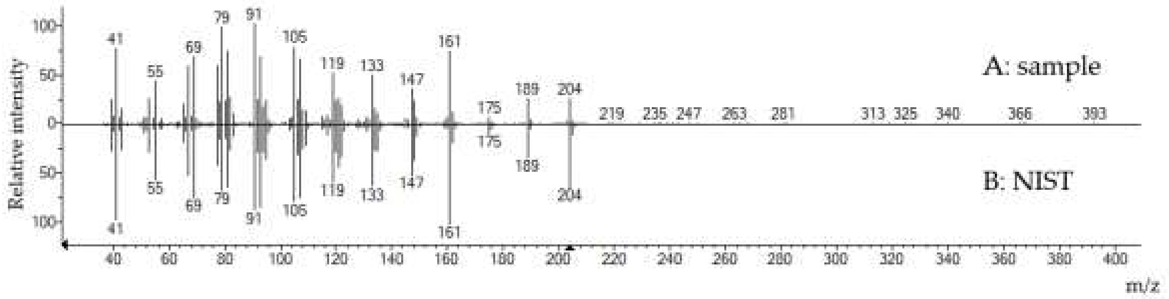

Figure S10 An example of comparing the mass spectrum of allo-aromadendrene (Rt = 32.18 min EO) in *Pterodon emarginatus* samples (panel A) and the NIST database (panel B). Match and Reverse Match exceeded 890 in all samples.

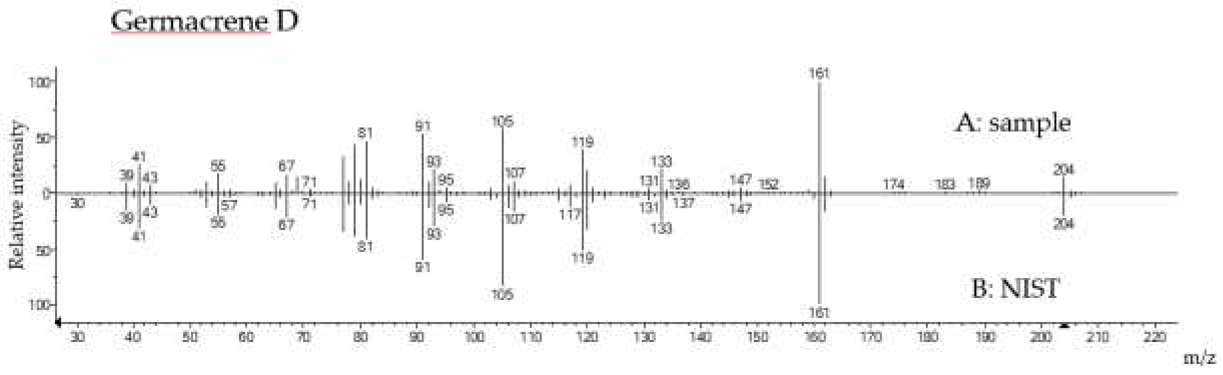

Figure S11 An example of comparing the mass spectrum of germacrene D (Rt = 33.54 min EO) in *Pterodon emarginatus* samples (panel A) and the NIST database (panel B). Match and Reverse Match exceeded 890 in all samples.

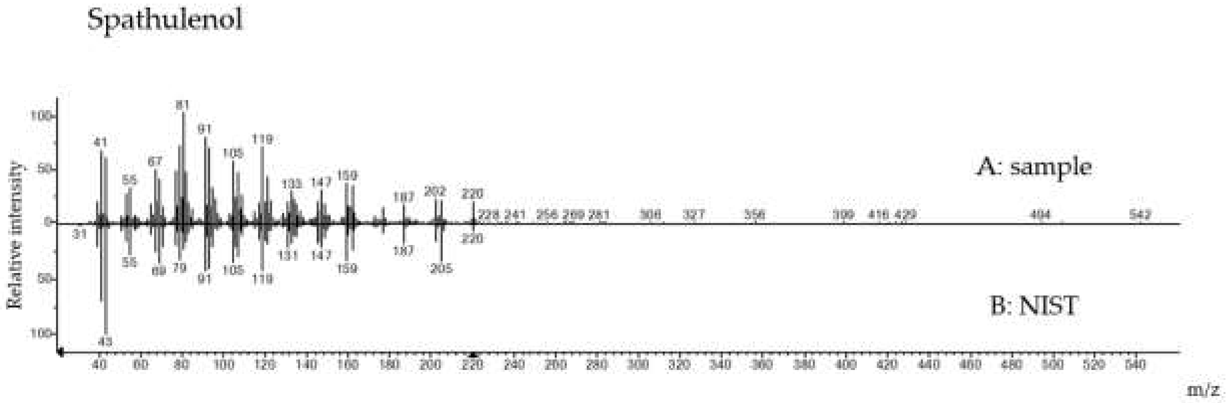

Figure S12 An example of comparing the mass spectrum of spathulenol D (Rt = 36.71 min EO) in *Pterodon emarginatus* samples (panel A) and the NIST database (panel B). Match and Reverse Match exceeded 890 in all samples.
